# Ole e 15 and its human counterpart -PPIA- chimeras reveal an heterogeneous IgE response in olive pollen allergic patients

**DOI:** 10.1038/s41598-019-51005-2

**Published:** 2019-10-21

**Authors:** Pablo San Segundo-Acosta, Carmen Oeo-Santos, Ana Navas, Aurora Jurado, Mayte Villalba, Rodrigo Barderas

**Affiliations:** 1 Departamento de Bioquímica y Biología Molecular, Facultad de Ciencias Químicas, Universidad Complutense de Madrid, E-28040 Madrid, Spain; 2 Hospital Universitario Reina Sofía de Córdoba, E-14004 Córdoba, Spain; 3 Chronic Disease Programme (UFIEC), Instituto de Salud Carlos III, Majadahonda, E-28220 Madrid Spain

**Keywords:** Immunology, Immunological disorders, Allergy

## Abstract

Olive pollen is a major cause of immunoglobulin E (IgE)-mediated allergy in Mediterranean countries. It is expected to become a worldwide leading allergenic source because olive cultivation is increasing in many countries. Ole e 15 belongs to the cyclophilin pan-allergen family, which includes highly cross-reactive allergens from non-related plant, animal and mold species. Here, the amino acid differences between Ole e 15 and its weak cross-reactive human homolog PPIA were grafted onto Ole e 15 to assess the contribution of specific surface areas to the IgE-binding. Eight Ole e 15-PPIA chimeras were produced in *E*. *coli*, purified and tested with 20 sera from Ole e 15-sensitized patients with olive pollen allergy by ELISA experiments. The contribution of linear epitopes was analyzed using twelve overlapping peptides spanning the entire Ole e 15 sequence. All the patients displayed a diverse reduction of the IgE-reactivity to the chimeras, revealing a highly polyclonal and patient-specific response to Ole e 15. IgE-epitopes are distributed across the entire Ole e 15 surface. Two main surface areas containing relevant conformational epitopes have been characterized. This is the first study to identify important IgE-binding regions on the surface of an allergenic cyclophilin.

## Introduction

Allergy is an immunoglobulin E (IgE)-mediated hypersensitivity which has become an important health problem in industrialized countries, affecting around 30% of their population. House dust mites and grass pollen are the most important aeroallergen sources^1,2^. However, in some regions, a higher prevalence of sensitization to specific local sources can be reached. Cultivated olive tree (*Olea europaea* ssp. *europaea* var. *europaea*) pollen is one of the main causes of pollinosis in the Mediterranean basin, being the main sensitizer in those areas where it is extensively cultivated^3^. Moreover, due to the continuous promotion of the Mediterranean diet, there is a rising demand for oil and olive fruit production worldwide, which will highly increase the number of olive trees and as a consequence, the number of allergic patients to this pollen^4^. To date, a total of fifteen olive allergens (Ole e 1 to 15) have been identified^5–7^. Besides, an extensive proteomic profiling of olive pollen has been recently reported, making this important allergenic source one of the best characterized^8^.

About 13% of the olive pollen allergic patients sera present IgE against Ole e 15, an allergen belonging to the group of cyclophilins. These proteins normally exhibit peptidyl-prolyl *cis-trans* isomerase activity and are part of the protein superfamily of immunophilins^9,10^. Due to their essential role in protein folding, cyclophilins are present in the cells of all organisms, and have been described as allergens in animals^11^, molds^12–14^ and plants^15–17^. Importantly, the amino acid sequence similarity among cyclophilins from unrelated species is high, and they could be considered pan-allergens due to their wide range of cross-reactivity^13^. Allergenic mold cyclophilins Mala s 6, Asp f 11 and Asp f 27 from *Malassezia sympodialis* and *Aspergillus fumigatus*, are major allergens whose structure and immunological properties have been extensively studied, showing *in vitro* and *in vivo* cross-reactivity between them and also with their human homologs peptidyl-prolyl *cis*-*trans* isomerases A (PPIA) and B (PPIB)^13,18,19^. Ole e 15, as well as other plant pollen allergenic cyclophilins, like Bet v 7 from birch and Cat r 1 from periwinkle, belong to the subgroup of divergent cyclophilins, which present a conserved Glu83 residue, two invariable cysteines and an additional stretch of seven amino acids with the consensus sequence XXGKXLH called divergent loop^20^. These allergens have also been shown to cross react with cyclophilins from plants^8,17,21^, animals (including human)^8^ and molds^17,21^.

The availability of structural information is essential to understand the cross-reactivity among different allergens, determine the nature and location of their IgE-binding epitopes and use this information to design safer allergen-specific immunotherapy strategies^22,23^. Multiple human, parasite and mold cyclophilins structures have been reported since the first PPIA crystal structure was determined^24,25^. Moreover, the structure of wheat, orange tree and periwinkle cyclophilins have been recently reported^17,26,27^, revealing important information about the distribution and accessibility of amino acids across the protein surface, and the regulatory function of specific structural elements, such as the divergent loop.

IgE-binding epitopes of most aeroallergens are mainly conformational and strongly dependent on the native allergen conformation^28–31^. This makes the analysis of epitopes a challenge, and only a few conformational IgE epitopes have been structurally solved using nuclear magnetic resonance (NMR) or X-ray crystallography^32–34^. Nevertheless, there are other strategies to identify specific IgE-binding surface areas, such as the use of random-peptide libraries mimicking real epitopes^23^, the design of point amino acid mutations to generate hypoallergenic variants^35^, epitope grafting^36^ and protein engineering to create chimeric proteins of the allergen and homologous proteins^37,38^. Although three main linear B-cell epitopes responsible for the cross-reactivity between mold cyclophilins and PPIA have been predicted by *in silico* analysis^39^, there is no experimental information available about the relevant epitopes of these allergens. Here, we aimed to identify the contribution of specific areas of Ole e 15 to the IgE-binding. After *in vitro* assessment of Ole e 15 and PPIA cross-reactivity, and *in silico* structure comparison and calculation of solvent-accessible surface areas, eight chimeras carrying specific regions from PPIA were designed using Ole e 15 as scaffold. Their IgE-binding capacity was assessed by means of ELISA and inhibition ELISA experiments and thus, relevant IgE-binding regions of Ole e 15 were identified.

## Results

### *In-vitro* Ole e 15-PPIA IgE cross-reactivity analysis and design of Ole e 15-PPIA chimeras

Six sera from olive pollen allergic patients reaching OD_492nm_ values higher than 0.4 when measuring IgE-binding to Ole e 15 by ELISA were tested with PPIA (Supplementary Fig. S1). Only serum 10 an 12 reached positive but low OD_492nm_ values when they were ten-fold diluted (Supplementary Fig. S1a). Inhibition IgE ELISA with serum 12 (Supplementary Fig. S1b) confirmed the previous results, with PPIA reaching inhibition values from 28% to 58% at concentrations at which Ole e 15 reached complete inhibition (4 μg/mL to 400 μg/mL).

**Figure 1:**
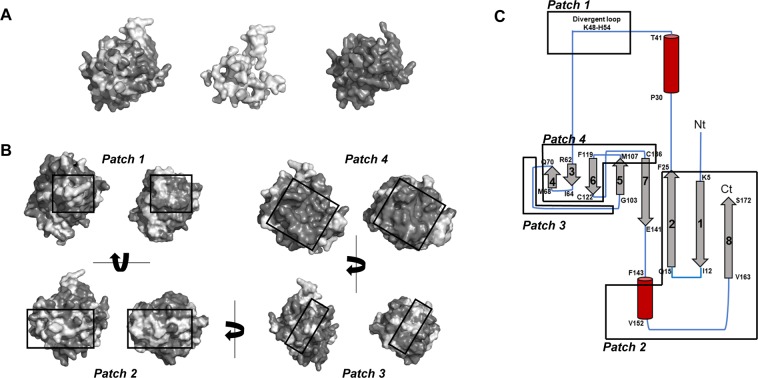
Structure comparison between Ole e 15 and human PPIA. (**A**) Modeled molecular surface of Ole e 15 (left). Light and dark gray colors indicate different and conserved amino acids between Ole e 15 and PPIA, respectively. The isolated surfaces of different (middle) and conserved (right) amino acids are also shown. (**B**) Modeled molecular surface pairs of Ole e 15 (left) and PPIA (right) for structure comparison. Three patches (*Patch 1*, *2 and 3*) containing most of the different amino acids between both molecules, and a fourth patch (*Patch 4*) with conserved amino acids were found. All patches are framed. Light and dark gray colors indicate different and conserved amino acids between Ole e 15 and PPIA, respectively. (**A**,**B**) All 3D-models were visualized using PyMOL 2.3 (https://pymol.org/2/). (**C**) Topological diagram showing the secondary elements of the predicted structure of Ole e 15. *Patch 1* comprises the amino acid stretch E43 to A59, including the divergent loop; *Patch 2* comprises the N-terminal M1 to A26, and the C-terminal Q138 to S172; *Patch 3* comprises A76 to G101; and *Patch 4* comprises H61 to T75 and M107 to V134. *Gray arrow*, β-strand; *red cylinder*, α-helix; *blue straight lines*, β-turns and random coil structures.

**Figure 2:**
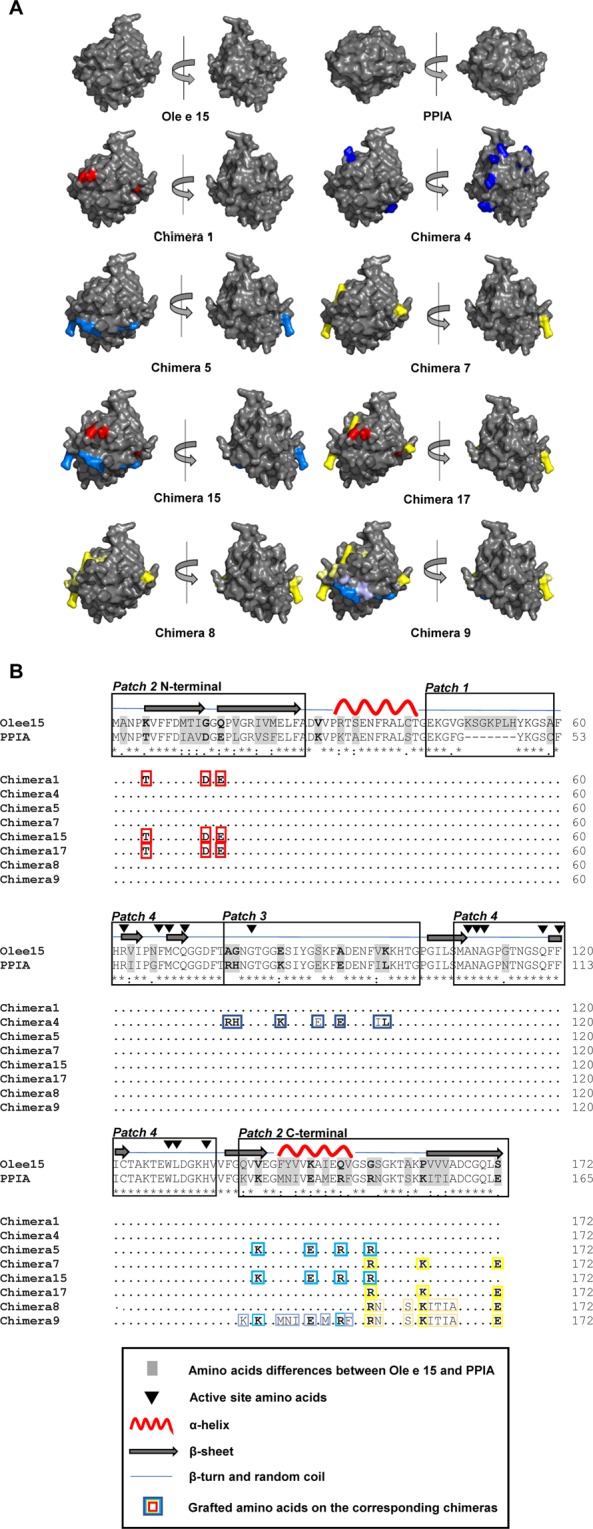
Modeled molecular surface and sequence and comparison between Ole e 15, PPIA and Ole e 15-PPIA chimeras. (**A**) Front and back (turned 180° on the vertical axis) modeled surfaces of Ole e 15, PPIA and chimeric Ole e 15-PPIA proteins. Grafted amino acids on the chimeras are colored. All 3D-models were visualized using PyMOL 2.3 (https://pymol.org/2/). (**B**) Amino acid sequence alignment of Ole e 15, PPIA and the chimeras. Amino acid differences between Ole e 15 and PPIA are shaded in gray. Amino acid differences with a change in the SASA value higher than 30 Å^2^ or higher than 10 Å^2^ but with changes in the amino acid charge state are shown in bold. PPIA amino acids grafted on the chimeras are indicated and framed with the same color as represented in (**A**). (*), fully conserved residues; (:), amino acids with groups of strongly similar properties; (.), amino acids with groups of weakly similar properties.

To find an explanation for this low-degree of IgE cross-reactivity in spite of the high sequence identity shared, the amino acid sequences and 3D-structures of Ole e 15 and PPIA were compared to identify their main differences (Figs 1 and 2, and Supplementary Fig. S2). Three surface patches comprising the amino acid stretches with the majority of amino acid differences between both molecules were identified: *Patch 1* (Ole e 15 stretch E43 to A59: divergent loop and surrounding residues), *Patch* 2 (Ole e 15 stretch M1 to A26: N-terminal β-strands β1 and β2, and surrounding residues; and stretch Q138 to S172: C-terminal β-strand β8, α-helix α2 and the connecting loop), and *Patch* 3 (Ole e 15 stretch A76 to G101: region connecting β-strand β4 to β5). A fourth patch corresponding to the most conserved surface region, including the active site, was also found (*Patch 4*, Ole e 15 stretch H61 to T75 and M107 to V134: β-strands β3, β4 and β7, and the loops connecting β-strands β3 to β4, β6 to β7, and β7 to β8). The main exposed amino acids of each patch (relative value to the isolated individual amino acid SASA higher than 25%) are described in Supplementary Fig. S2. The calculated Ole e 15 SASA per residue revealed a total SASA value of 14282.87 Å^2^, with values of 1343.81 (9.4%), 3222.82 (22.6%), 2219.88 (15.85%) and 2472.77 Å^2^ (17.3%) for the amino acid stretches included in Patches 1, 2, 3 and 4, respectively (Supplementary Table S1). Based on this information, nine protein chimeras were designed as described in the Material and Methods section.

### Biochemical characterization of the recombinant proteins

**Figure 3:**
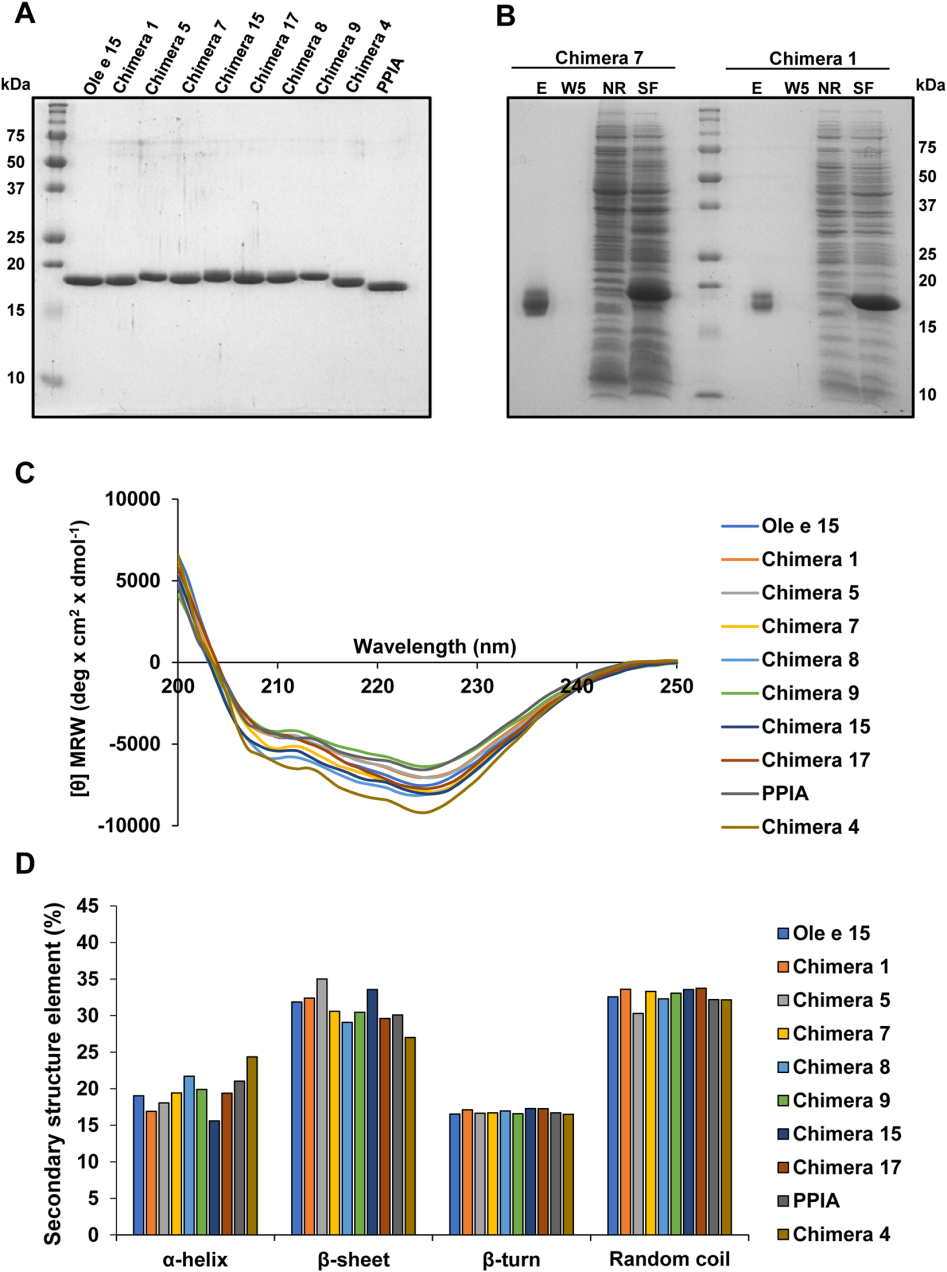
Biochemical characterization of Ole e 15-PPIA chimeras. (**A**) Coomassie Blue staining of 1 μg of the purified chimeras after 15% SDS-PAGE under reducing conditions. (**B**) Analysis of the purification process of Chimeras 1 and 7 by Coomassie Blue staining after 15% SDS-PAGE of fractions obtained from different purification steps. SF, bacterial soluble lysis fraction; NR, not retained proteins; W5, fifth Ni-NTA agarose wash; E, eluted protein (1 μg, not reduced). (**C**) Circular dichroism spectra of Ole e 15, PPIA and the chimeras. (**D**) Bar-graph showing percentage values of each secondary structure elements after spectra deconvolution using the CDNN software.

Ole e 15, PPIA and all protein chimeras were successfully expressed and purified from the soluble fractions of *E*. *coli* lysates (Fig. 3A,B), except the chimera lacking the Ole e 15 divergent loop (Chimera 3), which was expressed in inclusion bodies and could not be purified as a folded protein. Under non-reducing conditions, the purified proteins ran as two very close bands (Fig. 3B), as it has also been described for other cyclophilins^19,26^. CD spectroscopy was used to confirm a similar folding and the preservation of the structural integrity among Ole e 15, PPIA and the chimeras. All of them showed almost identical spectra, with barely observed changes at the secondary structure elements when spectra deconvolutions were performed (Fig. 3C,D).

The structure integrity of the recombinant proteins was further confirmed by ELISA experiments with an Ole e 15-specific pAb (Supplementary Fig. S3). ELISA experiments showed that all chimeras displayed a higher IgG-binding capacity than PPIA, with all of them, except Chimera 4, reaching OD_492nm_ values highly similar to Ole e 15 (Supplementary Fig. S3a). These results were further confirmed by inhibition ELISA. All protein chimeras, except Chimera 4, reached inhibition values similar to Ole e 15 (Supplementary Fig. S3b).

### Serum IgE-recognition patterns to Ole e 15 are highly diverse among olive pollen allergic patients

**Figure 4:**
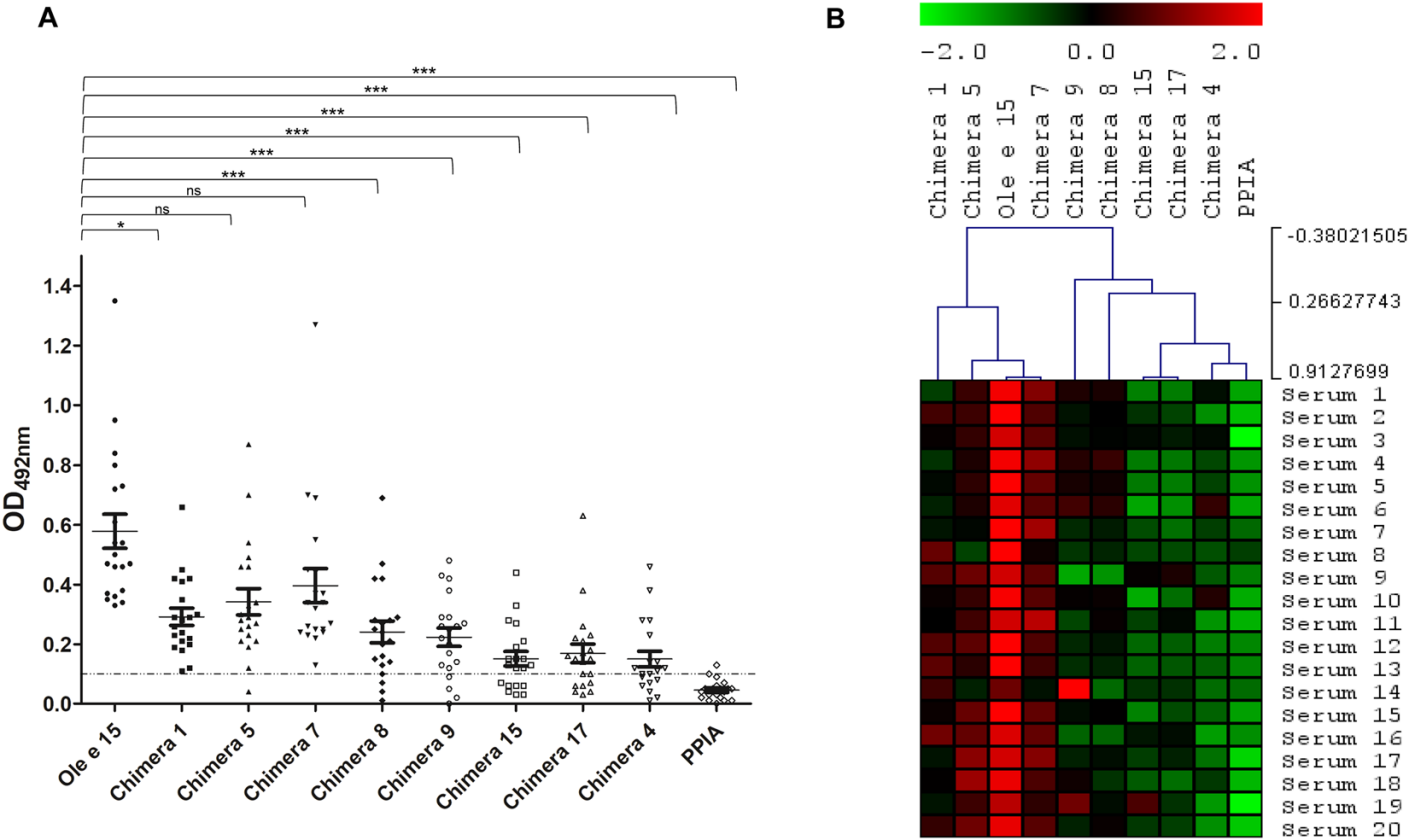
IgE-binding of Ole e 15, PPIA and Ole e 15-PPIA chimeras. (**A**) Scatter dot-plot representing the IgE-reactivity of the twenty patients to Ole e 15, PPIA and the chimeras assessed by ELISA. Results are shown as OD_492nm_ values (arbitrary units). *Horizontal bars* represent the arithmetic mean. *Vertical bars* represent the standard error of the mean (±SEM, *error bars* for duplicates). The *dashed line* represents the cut-off level of IgE-binding. *P < 0.05; ***P < 0.001, ns (not significant). (**B**) Visualization of the normalized results per patient in OD in (**A**) by hierarchical clustering analysis performed with MultiExperiment Viewer (MeV). Red, higher IgE levels. Green, low or nor IgE levels.

The IgE-binding capacity of Ole e 15, PPIA and the protein chimeras was assessed by ELISA (Fig. 4 and Supplementary Tables S2 and S3). Specific IgE-binding to Ole e 15 could be detected for the 20 used serum samples, whereas bound IgEs to PPIA could only be detected in the serum of two patients (Serum 10 and 12). Statistical analysis revealed a significant loss of IgE-recognition between Ole e 15, the protein chimeras and PPIA (Friedman test p-value lower than 0.001, mean OD for PPIA 0.05, mean ODs for protein chimeras 0.12–0.34, Supplementary Table S2). Significant differences between Ole e 15 and Chimera 1 (Dunn’s test p-value lower than 0.05), and Chimeras 8, 9, 4, 15 and 17 (Dunn’s test p-value lower than 0.001), but not significant when comparing to Chimeras 5 y 7 (Dunn’s test p-value higher than 0.05), were observed (Fig. 4).

Next, the different IgE-recognition profiles of each patient were examined. At least six different groups of patients could be distinguished according to their IgE recognition patterns (Supplementary Table S3). Importantly, all patients showed a decrease in the IgE-recognition capacity to protein chimeras compared to Ole e 15. The highest loss of IgE-recognition was mainly caused by the grafted regions on Chimera 4, and Chimeras 15 and 17 (Patterns 1, 2 and 4, 13 patients, 65%). Interestingly, some patients (Pattern 3, 3 patients, 15%) showed a higher loss against Chimeras 8 and 9, whose grafted regions correspond only to the C-terminal region of *Patch 2*. Other patients (Pattern 6, 2 patients, 10%) did not show a loss of IgE-recognition as high as other patients.

### The loss of Ole e 15-specific IgE-binding to the grafted PPIA regions on the protein chimeras is confirmed by inhibition ELISA

**Figure 5:**
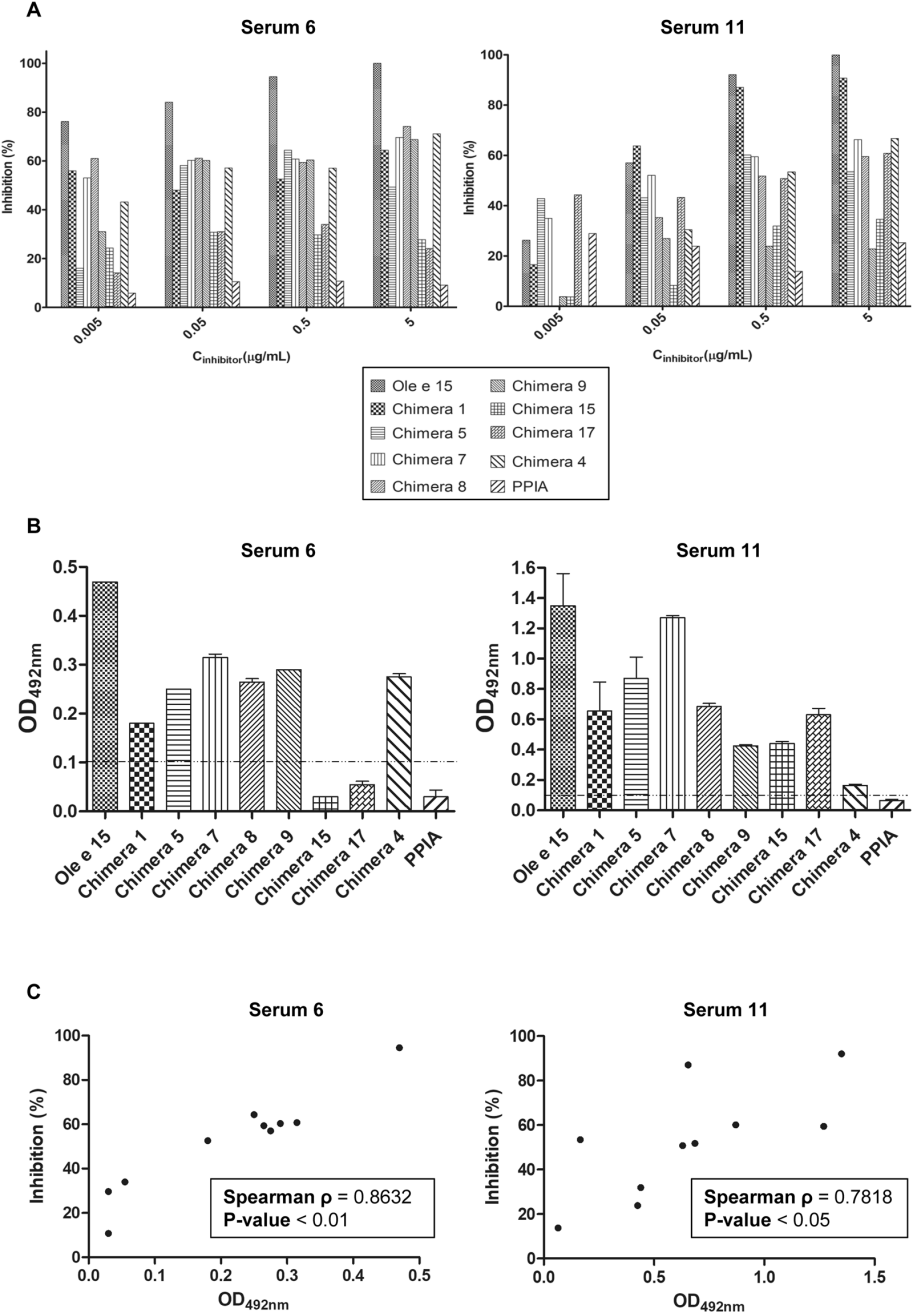
Immunological characterization of the IgE-response to Ole e 15, PPIA and Ole e 15-PPIA chimeras of two representative olive pollen allergic patients. (**A**) Bar graphs showing inhibition values of IgE-binding to immobilized Ole e 15 assesed by ELISA after serum preincubation with Ole e 15, PPIA or the Ole e 15-PPIA chimeras. (**B**) Bar graphs representing the mean OD_492nm_ (±SD) values obtained by ELISA for the assessment of the IgE-reactivity of serum samples 6 and 11. The *dashed line* represents the cut-off level of IgE-binding. (**C**) Two-tailed Spearman correlation analysis at 95% confidence interval between the obtained ELISA OD_492nm_ values and the IgE-inhibition values at inhibitor concentrations of 0.5 μg/mL. *Spearman ρ*, Spearman correlation coefficient.

The specific loss of IgE-binding capacity by the protein chimeras was further confirmed by inhibition ELISA experiments, in which Ole e 15 was coated and the chimeras and PPIA were used as inhibitors in the liquid phase. Results obtained from serum 6 and 11, for which inhibition experiments using all chimeras were performed, are shown in Fig. 5. Further results were obtained for different serum samples with representative chimeras (Supplementary Fig. S4). In all cases Ole e 15 reached inhibition values near 95–100% at 5 μg/mL, whereas values for protein chimeras were variable and serum specific (Fig. 5A and Supplementary Fig. S4), ranging from 11% to 90%. The maximum inhibition value reached by PPIA was 25% (serum 11). The obtained OD values by ELISA with serum 6 and 11 (Fig. 5B) were compared to the inhibition values. A significant (p-values lower than 0.05) and high correlation (Spearman ρ values of 0.85 and 0.78, respectively) was found (Fig. 5C).

### Assessment of the IgG- and IgE-binding capacity of Ole e 15-derived peptides

Finally, to analyze the IgG- and IgE-binding to linear amino acid stretches on the surface of Ole e 15, thirteen overlapping peptides covering the entire Ole e 15 sequence were produced at the C-terminal end of 6 × His-HaloTag constructs (Supplementary Fig. S5). Linear epitope mapping of the Ole e 15-specific pAb was performed by ELISA using immobilized peptides. Regions covered by peptides 6, 7, 8 and 9 (*Patch 3* and near residues), and also 12 and 13 (*Patch 2*, C-terminal region), were the most IgG-immunoreactive (Supplementary Fig. S6a). The IgE-binding capacity of peptides corresponding to the amino acid sequences of *Patch 1* (Peptides 4 and 5), *Patch 2* (Peptides 1, 2, 12 and 13) and *Patch 3* (Peptides 7 and 8) was assessed by inhibition ELISA with four representative serum samples (Supplementary Fig. S6b). Little or no IgE-inhibition was achieved by the peptides, except for Peptides 4 and 5, which reached 63.1% inhibition of the serum 3 IgE-reactivity to immobilized Ole e 15.

Collectively, these results confirmed that most of the Ole e 15 IgE epitopes are non-linear conformational epitopes.

## Discussion

In this study, we aimed at identifying relevant IgE-binding regions to Ole e 15 by grafting amino acids from specific surface areas of human PPIA, after confirming the lower PPIA capacity of binding Ole e 15 specific-IgEs from olive pollen allergic patients. Then, we assessed the potential loss of IgE-binding of the resulting Ole e 15-PPIA chimeras. Although similar approaches have already been used with other allergens like Bet v 1^37,40^, Mal d 1^36^, Ole e 1^38^, Ves v 5^41^ and Pen a 1^42^, we are the first to report information on relevant IgE-binding surface areas of an allergenic plant cyclophilin.

The sequence and surface comparison between PPIA and Ole e 15 revealed that the amino acid changes between both molecules, although only comprising 21.5% and 24.6% of the Ole e 15 and PPIA total SASA, respectively, were distributed over the entire surface (Fig. 1A). Therefore, these changes could be responsible for the loss of the IgE-binding capacity of several surface areas which in Ole e 15 correspond to potential main epitopes, explaining the low seroreactivity of PPIA (Supplementary Fig. S1).

A typical conformational IgG or IgE protein epitope comprises surface areas of around 700~1000 Å^2^, as revealed by X-ray crystallography studies on antibody-antigen complexes^34,43,44^. Three main Ole e 15 surface patches were identified with areas wide enough to be epitopes, and containing most of the amino acid differences between both molecules (Fig. 1B,C, Supplementary Fig. S2 and Supplementary Table S1). Then, PPIA amino acids were grafted onto these patches, generating eight chimeric proteins, to assess the contribution of the substituted regions to the IgE-binding to Ole e 15. An essential prerequisite to obtain reliable information from the grafted areas is the conservation of an equivalent protein folding. Thus, CD spectroscopy was used to analyze the secondary structure of Ole e 15, PPIA and chimeric proteins. A highly-similar spectra and an almost equivalent proportion of secondary structure elements were found (Fig. 3). The conservation of the cyclophilin folding was also confirmed by ELISA and inhibition ELISA experiments with an Ole e 15-specific pAb (Supplementary Fig. S3). All proteins showed IgG-binding capacity, revealing common epitopes between Ole e 15 and PPIA (Supplementary Fig. S3a). Moreover, all chimeras except Chimera 4 showed OD and inhibition values equivalent to those of Ole e 15 (Supplementary Fig. S3b). These results suggested that the mutated amino acids of Chimera 4 were essential for the pAb-binding to at least one important epitope composed by amino acids between D75 to A110, as revealed by the pAb-linear epitope mapping (Supplementary Fig. S6a). The correct folding was further confirmed after SDS-PAGE under non-reducing conditions and Coomassie Blue staining, which showed that purified proteins ran as a lower band corresponding to the reduced state of the protein and a close upper band representing an oxidized form with an intramolecular disulfide bond (Fig. 3B). This bond, formed between C40 and C168 residues, which are very close in the native structure, has been shown to be part of a 2-cysteine redox mechanism controlling enzyme activity^19,26^.

Among the three identified surface patches with most of the amino acid changes between Ole e 15 and PPIA, the *Patch 2* covered the widest SASA (3222.82 Å^2^), contained three defined sequence stretches (N-terminal M1 to A26, inner C-terminal Q138 to G155 and outer C-terminal G155 to S172 regions) and accumulated the highest number of amino acid changes between Ole e 15 and PPIA. Numerous studies describe that antibody-antigen interactions mainly depend on charged, polar or aromatic epitope residues with a high SASA^38,45,46^. Then, assuming that a high change in the SASA value or a change in the charge state of amino acids would most probably alter the potential IgE-binding to each region, only PPIA amino acids meeting a set of criteria (see Materials and Methods section) were grafted onto Ole e 15 to create Chimeras 1, 5 and 7 (Table 1 and Fig. 2). Interestingly, all serum samples showed a loss of seroreactivity against these three chimeric proteins, confirming that the mutated amino acids, very close in the 3D-structure, were part of one or more conformational IgE-binding epitopes located on *Patch 2* (Fig. 2 and Supplementary Table S2). The loss of IgE-reactivity against the three chimeras was similar for some patients (Pattern 1, Supplementary Table S3), but in other cases the loss against Chimera 1 was higher (Pattern 2 patients, Supplementary Table S3), suggesting a patient-specific IgE-repertoire against *Patch 2*. For Pattern 1 patients, all the mutated amino acids might similarly contribute to the IgE-binding to a common conformational epitope, whereas in the case of Pattern 2 patients, the N-terminal residues might be more relevant to the IgE-binding to that epitope or be part of a different one.

**Table 1:** Mutated amino acids in the chimeric proteins.

Recombinant Ole e 15-PPIA* chimeras	
	Amino acid substitutions
Chimera 1^a^	K5T, G13D, Q15E
Chimera 3^b^	K48 to H54 deletion
Chimera 4^c^	A76R, G77H, E83K, S88E, A91E, V96I, K97L
Chimera 5^d^	V140K, K147E, Q151R, G155R
Chimera 7^e^	G155R, P162K, S172E
Chimera 8^f^	G155R, S156N, P162K, V163I, V164T, V165I, S172E
Chimera 9^g^	Q138K, V140K, K147E, Q151R, G155R, S156N, P162K, V163I, V164T, V165I, S172E
Chimera 15^h^	K5T, G13D, Q15E, V140K, K147E, Q151R, G155R
Chimera 17^i^	K5T, G13D, Q15E, G155R, P162K, S172E

*PPIA, human cyclophilin A (also known as CypA).

^a^N-terminal *Patch 2* (M1 to A26) mutations (only amino acids meeting the criteria on solvent exposure/charge state changes).

^b^Deletion of the divergent loop (Patch 1).

^c^Whole *Patch 3* (A76 to G101) mutations (all different amino acids were grafted).

^d^Inner C-terminal *Patch 2* (V140 to G155) mutations (only amino acids meeting the criteria).

^e^Outer C-terminal *Patch 2* region (G155 to S172) mutations (only amino acids meeting the criteria).

^f^Outer C-terminal *Patch 2* region mutations (all different amino acids were grafted).

^g^Whole *Patch 2* C-terminal region (Q138 to S172) mutations (all different amino acids were grafted).

^h^N-terminal and inner C-terminal *Patch 2* mutations (only amino acids meeting the criteria).

^i^N-terminal and outer C-terminal *Patch 2* mutations (only amino acids meeting the criteria).

Mutations at the N- and C-terminal regions were combined in Chimeras 15 and 17 to further analyze their contribution to the IgE-recognition of *Patch 2*. Both chimeras displayed a higher loss of IgE-reactivity than Chimeras 1, 5 and 7, and for some patients that loss was complete. These results confirmed that the mutated amino acids were part of at least one relevant epitope located on *Patch 2* and surrounding areas. Moreover, the contribution of the whole C-terminal *Patch 2* region (Q138 to S172) to IgE-binding was further studied using Chimeras 8 and 9. For Pattern 2 and Pattern 6 patients, the loss of seroreactivity by Chimeras 8 and 9 was similar to that caused by Chimeras 5 and 7, suggesting that the amino acid mutations of Chimeras 5 and 7 were sufficient to provoke the loss of IgE-binding. On the other hand, for Pattern 1 or Pattern 3 patients, those amino acids mutations were not sufficient, and the exclusive mutations of Chimeras 8 and 9 provoked a higher loss, which further supported the idea that these patients present a different IgE-repertoire against this region. Besides, the fact that Chimeras 8 and 9 showed a similar loss of IgE-reactivity by most patients indicated that the amino acid mutations of Chimera 8 were sufficient to lower IgE-binding.

For Chimera 4, seven amino acids were grafted from PPIA onto Ole e 15 to analyze the role of *Patch 3* in IgE-binding (Table 1 and Fig. 2). Interestingly, most of the serum samples showed a reduced IgE-binding capacity to this chimera compared to Ole e 15, suggesting this region was part of a relevant epitope. The sequence belonging to this patch, which contains the amino acids of the regulatory catalytic loop^26^, had the highest relative SASA per amino acid (Supplementary Table S1). Besides, five of the seven mutated amino acids in this region met the criteria on changes in the SASA value and charge state. Thus, the number and nature of these amino acid changes might explain why the IgE-reactivity of Chimera 4 was also the lowest of all the chimeras.

Several IgE-binding studies of Bet v 1^36,37^, Der p 2^47,48^ and other allergens^49,50^ have revealed that the allergen-specific IgE-repertoire can be very complex in terms of IgE-clonality, affinity and concentration. Besides, it is well-known that these parameters affect effector cell degranulation, which is only triggered in the presence of at least two IgE-clones binding non-overlapping epitopes on the allergen surface^51^. This is consistent with our results, where at least six different serum recognition patterns against the chimeric proteins could be identified and all patients lost IgE-recognition to a different extent when amino acids from *Patch 2* and *Patch 3*, belonging to at least two different epitopes, were mutated. Interestingly, it has been proposed that every region of the solvent exposed surface of a protein can be potentially immunogenic and then, part of a B-cell epitope, although some epitopes are more recognized than others^52,53^. In fact, a recent study showed that 20 out of 64 Bet v 1-allergic patients tested presented IgEs against at least four well-defined regions on the Bet v 1 surface after they were grafted onto the homologous low IgE-binding allergen Api g 1^37^.

Other two patches on the surface of Ole e 15 (*Patch 1* and *Patch 4*, Fig. 1 and Supplementary Fig. S2) with sufficient areas to contain potential epitopes were identified. Regarding *Patch 1*, an Ole e 15 mutant lacking the divergent loop amino acid sequence was designed (Table 1), but it could not be produced as a folded protein. However, in a previous study in which an equivalent Cat r 1 mutant could be produced, no loss of its IgE-binding capacity could be detected^17^. Here, after using two peptides containing the loop sequence and surrounding residues as solution inhibitors by ELISA, we only observed high inhibition values in one of the four serum samples tested (Supplementary Fig. S6b). Interestingly, this patient did not show a high reduction of the IgE-reactivity compared to other patients (recognition pattern 6), so *Patch 1* residues might be part of a relevant IgE-binding region for some patients. Other peptides were also used to analyze the potential contribution of sequential amino acid stretches in *Patch 2* and *Patch 3*, but no important inhibition values were reached, further suggesting that the amino acids of these regions were part of conformational epitopes. Nevertheless, the use of peptides gives limited information on the IgE-binding capacity of the represented regions, as they do not usually display the real topological structure of the native protein surface^31,53^. Thus, the contribution of sequential stretches to the IgE-binding to these regions cannot be fully discarded, and further studies should be performed to determine whether *Patch 1* is a region containing amino acids belonging to a main epitope for olive pollen allergic patients.

The IgE-mediated cross-reactivity between allergens sharing high sequence identity with their human counterparts has been described for proteins like the pan-allergens profilins or the fungal allergens manganese superoxide dismutase, acidic ribosomal P2 protein and thioredoxin^54^. This cross-reactivity has also been demonstrated *in vitro* and *in vivo* between the fungal cyclophilins Mala s 6 and Asp f 11 and PPIA and PPIB, and seem to be especially relevant in patients suffering from allergic bronchopulmonary aspergillosis^19^. Here, only two patients showed IgE-reactivity against PPIA by ELISA, and very low inhibition values were reached using PPIA as inhibitor, revealing a weak *in vitro* cross-reactivity between Ole e 15 and PPIA among olive pollen allergic patients. Interestingly, the protein regions predicted *in silico* as the main epitopes accounting for this interspecies cross-reactivity belong to the active site region^39^. In Ole e 15, these regions are part of the *Patch 4*. Hence, the weak Ole e 15-PPIA cross-reactivity might be explained by the presence of IgE antibodies in low levels or with low affinity or clonality against this region. Nevertheless, it has been proven that the presence in the IgE-repertoire of only two non-overlapping allergen-specific antibodies, one with high affinity and another with very low affinity, is sufficient to produce functional high affinity IgE receptor (FcɛRI)/IgE/allergen complexes on the surface of effector cells^51^. Then, this *in vitro* cross reactivity, although weak, might be clinically relevant, and further studies to address it by *in vivo* experiments and to confirm the role of *Patch 4* as an IgE-binding region should be performed.

The identification of relevant IgE epitopes on the surface of allergens gives fundamental information about the generation of the IgE-repertoire by allergic patients and allows for a better prediction of cross-reactivity between allergens by using epitope sequence similarity rather than whole allergen sequence^53^. Besides, it is important for the design of hypoallergenic variants with low capacity of effector cell activation for allergen-specific immunotherapy^31,55^. Here, Chimeras 15, 17 and 4 displayed a very reduced IgE-binding capacity compared to Ole e 15 and their suitability as hypoallergenic variants should be addressed by basophil activation tests and T-cell reactivity experiments.

## Conclusion

In summary, this study shows that there is a weak but relevant *in vitro* cross-reactivity between PPIA and Ole e 15 and that the Ole e 15-specific IgE-response is highly polyclonal and patient specific. Two main IgE-binding regions on the surface of Ole e 15 have been identified after the immunological characterization of eight Ole e 15-PPIA chimeras displaying lower IgE-binding capacities than Ole e 15. Thus, we demonstrate that grafting specific areas from a low IgE-binding homolog like PPIA is useful to identify the main IgE-binding sites of an allergen.

## Materials and Methods

### Patients and control subjects

Twenty sera from olive pollen allergic patients obtained by standard operating procedures from two different Spanish populations (Córdoba and Madrid) were used according to their positive olive pollen skin prick test (SPT), presence of IgE antibodies against olive pollen extract as measured by ImmunoCAP 250 (Thermo Scientific, Uppsala, Sweden) or ELISA, and presence of specific IgE against recombinant Ole e 15 (Ole e 15) as determined by ELISA. SPT was performed according to standard procedures. A wheal diameter lower than 3 mm was considered negative. Values higher than 0.35 kU/L by ImmunoCAP, or higher than 0.1 optical density at 492 nm (OD_492 nm_, arbitrary units) by ELISA were considered positive. All patients included in this study reported an immediate IgE-mediated allergic reaction and a positive SPT with olive pollen.

The Institutional Ethical Review Boards of the Complutense University of Madrid, ISCIII (CEI PI 49) and Reina Sofia University Hospital (ref. 3033) approved this study involving allergic patients. All samples were handled anonymously according to the ethical and legal guidelines of the Complutense University of Madrid and Reina Sofia University Hospital after approval of the Ethical Review Boards of these institutions. All subjects in the study gave their written informed consent to participate and all experiments were performed in accordance with relevant guidelines and regulations. All patients presented asthma or rhinoconjunctivitis. Detailed demographic and clinical information of the olive pollen allergic patients is depicted in Supplementary Table S4. Three non-atopic subjects were used as controls.

### Ole e 15 and PPIA structure comparison and design of Ole e 15-PPIA chimeras

A three-dimensional (3D)-model of Ole e 15 was obtained with the ExPASy homology-modelling server Swiss-Model^56^ using the NMR solution ligand-free structure of the allergenic plant cyclophilin Cat r 1 as template (PDB: 2mc9, 86% sequence identity and 92% similarity with Ole e 15). For PPIA, the NMR solution structure (PDB: 1OCA) was used. Three-dimensional structures were visualized using PyMOL 2.3 (Schrödinger, LCC, New York) and amino acid sequences were aligned using Clustal Omega^57^. Sequence and structure comparison allowed for the identification of four main surface patches gathering most of the different (*Patches 1*, *2* and *3*) or the conserved (*Patch 4*) amino acids between Ole e 15 and PPIA (see Figs 1 and 2, and Supplementary Fig. S2). Then, solvent-accessible surface areas (SASAs) per residue from both molecules were calculated using Parameter OPtimized Surfaces (POPS)^58^, and the SASAs of the changing amino acids were compared. All protein chimeras were created by grafting amino acids from PPIA onto Ole e 15. Amino acid pairs belonging to *Patch 2*, and meeting the following criteria, were considered for grafting in the case of Chimeras 1, 5 and 7: a change of SASA value higher than 30 Å^2^, or a change of SASA value higher than 10 Å^2^ but with a change in the potential charge state of the amino acid (Table 1, Fig. 2, and Supplementary Table S1). Thus, Chimera 1 was designed by mutating three amino acids in the *Patch 2* N-terminal region (M1 to A26), and Chimeras 5 and 7, by mutating four amino acids in the *Patch 2* inner C-terminal region (Q138 to G155) and three amino acids in the *Patch 2* outer C -terminal region (G155 to S172), respectively. Chimeras 15 and 17 were designed as a combination of the mutations in Chimeras 1 and 5, and Chimeras 1 and 7. For the rest of the chimeras, the above criteria were not applied. Chimera 4 was designed by changing all the corresponding amino acids in *Patch 3*; Chimera 8, all amino acids in the *Patch 2* inner C-terminal region; and Chimera 9, all amino acids in the *Patch 2* (inner C-terminal and outer C-terminal regions, Q138 to S172). A deletion mutant (Chimera 3) was also designed by removing the amino acids of the Ole e 15 divergent loop (K48 to H54 in *Patch 1*). All amino acid changes are summarized in Table 1.

### Cloning, expression and purification of recombinant proteins

Overlapping oligonucleotides were designed to generate the chimera-coding DNA fragments by PCR amplification. pET28a-derived expression vectors containing Ole e 15 and PPIA-coding sequences were used as templates. The obtained fragments were later assembled into expression vectors using NEBuilder HiFi DNA Assembly Mix (New England Biolabs, USA). Thirteen peptides of twenty four amino acids in length and with twelve amino acid overlaps covering the entire Ole e 15 amino acid sequence were also designed (Supplementary Fig. S5). Each DNA insert with the peptide coding sequence was generated by PCR-amplification of *att*B-site containing oligonucleotides overlapping by twenty nucleotides. Then, each insert was simultaneously cloned into pDONR221 and pDEST-HisHALO as donor and destination (expression) vectors, respectively, using single-step combined BP/LR Gateway reactions (Thermo Fisher Scientific, Waltham, MA, US)^59^. Oligonucleotides were purchased from Sigma-Aldrich (St.Louis, MO, US) (Supplementary Table S5). A random peptide (control peptide) with the sequence HGRIKQVCTKKQASSGVMLGDPNS was used as control.

Ole e 15, PPIA, protein chimeras and HaloTag-fused peptides were recombinantly produced in *E*. *coli* as N-terminal His_6_-tagged proteins. The pET28a/pDEST-HisHALO expression constructs were used to transform BL21 (DE3) *E*. *coli* cells. LB cultures of 10 mL with 30 μg/mL kanamycin containing the transformed cells were grown overnight and then, diluted ten times and grown until OD_600nm_ reached 0.7. Finally, protein expression was induced with 0.4 mM isopropyl thio-β-D-thiogalactoside (IPTG) and grown at 37 °C 230 rpm four hours (for HaloTag-fused peptides 1 to 11, 13 and control peptide), 30 °C 230 rpm for sixteen hours (for Ole e 15, PPIA and Chimeras 1, 5, 7, 8, 15, 17), or at 16 °C 230 rpm for forty eight hours (for Chimeras 4 and 9, and HaloTag-fused peptide 12). Then, the cultures were centrifuged at 6,000* × g* for twenty minutes at 4 °C. Proteins were purified by gravity-flow chromatography from the soluble fraction of cell lysates using Ni-NTA agarose (Qiagen, Hilden, Germany) following the manufacturer instructions with minor modifications. Briefly, cell pellets corresponding to 250 or 500 mL *E*. *coli* cultures were resuspended in 10 mL lysis buffer (300 mM NaCl, 1 mM PMSF, 50 mM phosphate buffer pH 7.0), distributed in 1 mL aliquots and disrupted by five cycles of subsequent freezing in liquid N_2_ for forty seconds and thawing at 42 °C in a water bath. Then, soluble fractions obtained by centrifugation at 6,000 × *g* for twenty minutes at 4 °C were incubated sixteen hours at 4 °C in a 15 mL conical centrifuge tubes with 500 μL Ni-NTA agarose previously pre-incubated three times with 5 mL lysis buffer for two minutes. After that, Ni-NTA agarose was washed five times for ten minutes at 4 °C with 5 mL wash buffer (10 mM imidazole, 300 mM NaCl, 50 mM phosphate buffer pH 7.0), recovering each time the resin by centrifugation at 4,000 × *g* for ten minutes at 4 °C. Proteins were then eluted with 5 mL elution buffer (250 mM imidazole, 300 mM NaCl, 50 mM phosphate buffer pH 7.0) for twenty minutes at 4 °C on rotation. To avoid the presence of traces of Ni-NTA agarose in the eluted fractions, a final centrifugation step was performed at 12,000 × *g* for five minutes at 4 °C. Finally, purified proteins were desalted onto PD10 desalting columns (GE Healthcare, Chicago, IL, US) with 150 mM ammonium bicarbonate, quantified, aliquoted, lyophilized and stored at −20 °C until use.

### Analytical methods, antibodies and physicochemical analysis

Purity of the recombinant proteins was analyzed by 15% SDS-PAGE and subsequent staining with Coomassie Blue R-250 (Sigma-Aldrich, St.Louis, MO, US). Theoretical molecular mass calculations were performed using unstained protein biomarkers SM0431 (Fermentas, Waltham, MA, US).

The rabbit polyclonal antiserum against Ole e 15 (Ole e 15-specific pAb) was previously obtained^8^.

Circular dichroism (CD) spectra of Ole e 15, PPIA and protein chimeras in 20 mM phosphate buffer pH 7.0, at 0.2 mg/mL and 20 °C were recorded in the far-UV (190–260 nm) on a J-715 spectropolarimeter (JASCO, Japan Spectroscopic Co., Tokyo, Japan) using a 0.1 cm optical-path quartz cuvette. The deconvolution of the obtained spectra was performed using the CDNN software (thirty three reference net spectra, complex CD spectrum). Finally, the obtained far UV spectra were baseline-subtracted and represented as mean residue molecular ellipticity.

Protein concentration of purified Ole e 15, PPIA and protein chimeras was calculated after amino acid analysis of 14 μg purified proteins in duplicates with a BioChrom 30 Amino Acid analyzer (Harvard Bioscience, Inc., Holliston, MA, US). Protein concentration of the purified HaloTag-fused peptides was calculated by measuring the absorbance at 280 nm using a DU-7 spectrometer (Beckman, Barcelona, Spain) after theoretical extinction coefficient calculation with the ProtParam tool from ExPASy.

### IgE and Ole e 15-specific pAb ELISA experiments

To detect IgE-binding by ELISA, high-binding microplates (Costar, Corning, New York, US) were coated overnight at 4 °C with 50 μL/well of 10 μg/mL Ole e 15, PPIA and protein chimeras in phosphate-buffered saline (PBS). After washing four times with 0.5% Tween 20-PBS, non-specific binding sites were blocked at 37 °C one hour with 100 μL/well 3% skimmed milk 0.1% Tween-PBS (blocking buffer). Then, 50 μL/well sera ten-fold diluted in blocking buffer were incubated in duplicate at 37 °C for two hours. After washing, bound IgEs were detected by incubating 50 μL/well 10^3^-fold blocking buffer-diluted horseradish-peroxidase-conjugated mouse anti-human IgE mAb (Southern Biotech, Birmingham, AL, US) one hour at 37 °C. Color was developed for thirty minutes with *o*-phenylenediamine chloride and H_2_O_2_ as substrates (Merck, Darmstadt, Germany), and the reaction was stopped with 1 N H_2_SO_4_. Absorbance was measured at 492 nm. Incubation with three sera from non-atopic individuals, and incubation with blocking buffer instead of serum (background), were used as controls of the assay. Normalized OD values (sample OD_492nm_-background) were considered positive when the cut-off absorbance value was higher than 0.1 (exceeding always the mean background by 3 SDs). For Ole e 15-specific pAb-ELISAs, plates were coated with 100 μL/well of 1 μg/mL proteins or 5 μg/mL fusion-peptides, in PBS. For Ole e 15, PPIA and protein chimeras titration curves, each well was incubated with 100 μL serial dilutions (from 10^4^ to 3 × 10^4^-fold) of Ole e 15-specific pAb in blocking buffer at 37 °C for one hour. To assess the recognition of the HaloTag fused-peptides, the antibody was 2.5 × 10^3^-fold diluted. Specific IgG-binding was detected using a horseradish peroxidase-labeled goat polyclonal antibody against rabbit IgG (Bio-Rad, Richmond, CA, USA).

### Inhibition ELISAs

For all inhibition ELISAs, procedures like coating, blocking and color development were performed as described above. For IgE-inhibition with Ole e 15, PPIA and chimeras, diluted sera (seven point five to twenty five-fold) were preincubated in duplicate for two hours at room temperature with ten-fold serial dilutions, from 0.005 to 5 μg/mL, or alternatively, 0.001 to 1 μg/mL, of the inhibitor proteins. For IgE-inhibition with HaloTag-fused peptides, diluted sera (fifteen to twenty five-fold) were preincubated with each peptide at 2 μM concentration.

Inhibition values were calculated with the following formula:

$${\rm{Inhibition}}( \% )=(1-{{\rm{OD}}}_{{\rm{inhibited}}}/{{\rm{OD}}}_{\mathrm{non}-\mathrm{inhibited}})\times 100$$

### Statistical analysis

The Friedman test and the Dunn’s post test (95% Confidence Interval (CI)) were performed to analyze whether the reduction of the IgE-recognition capacity of each serum against protein chimeras and PPIA in comparison to Ole e 15, was significant. Moreover, the relationship between the OD_492nm_ values obtained by ELISA and the inhibition experiments was analyzed using Spearman correlation (95% CI).

## Supplementary information

Supplementary Information

Supplementary Information

Supplementary Information

## Data Availability

The data are available as supplementary information.
