# Supplementary material for: Ole e 15 and its human counterpart -PPIA- chimeras reveal an heterogeneous IgE response in olive pollen allergic patients
Source: Sci Rep. 2019 Oct 21;9:15027. doi: 10.1038/s41598-019-51005-2 (PMC6803672; doi:10.1038/s41598-019-51005-2)
Supplement: Supplementary file 1 — Supplementary Information [file 41598_2019_51005_MOESM1_ESM.pdf]

Ole e 15 and its human counterpart -PPIA- chimeras reveal an heterogeneous IgE response to Ole e 15 in olive pollen allergic patients

Pablo San Segundo-Acosta[‡], Carmen Oeo-Santos[‡], Ana Navas[¶], Aurora Jurado[¶], Mayte Villalba[‡,*], Rodrigo Barderas[*,&]

[‡]Departamento de Bioquímica y Biología Molecular, Facultad de Ciencias Químicas, Universidad Complutense de Madrid, E-28040 Madrid, Spain, [¶]Hospital Universitario Reina Sofía de Córdoba, E-14004 Córdoba, Spain, [&]Chronic Disease Programme (UFIEC), Instituto de Salud Carlos III, Majadahonda E-28220, Madrid, Spain

[*]Co-senior authors. To whom correspondence should be addressed

CONTENTS **PAGE/S**

**Supplementary Table S1**	**Separate File**
**Supplementary Table S2**	**3-4**
**Supplementary Table S3**	**Separate File**
**Supplementary Table S4**	**5-6**
**Supplementary Table S5**	**7-8**
**Supplementary Fig. S1**	**9**
**Supplementary Fig. S2**	**10**
**Supplementary Fig. S3**	**11**
**Supplementary Fig. S4**	**12**
**Supplementary Fig. S5**	**13**
**Supplementary Fig. S6**	**14**

Supplementary Table S2. Raw indirect ELISA data obtained from the twenty individual olive pollen allergic patients and the three healthy subjects used in the study, and statistical analysis.

Serum	Duplicate mean of the measured OD values at 492 nm (raw data) after subtracting buffer control (background)								
	Ole e 15	Chimera 1	Chimera 5	Chimera 7	Chimera 8	Chimera 9	Chimera 15	Chimera 17	Chimera 4	PPIA
**Healthy subject 1**	0.06	0.01	0.02	0	0	0.01	0.01	0	0	0.06
**Healthy subject 2**	0.04	0.01	0.01	0.01	0	0.01	0.01	0.01	0.01	0
**Healthy subject 3**	0.03	0.01	0	0	0	0	0	0	0	0
**Serum 1**	0.95	0.28	0.54	0.7	0.47	0.48	0.13	0.14	0.38	0.05
**Serum 2**	0.37	0.21	0.21	0.22	0.16	0.14	0.12	0.1	0.04	0
**Serum 3**	0.47	0.29	0.33	0.37	0.28	0.27	0.28	0.26	0.28	0.02
**Serum 4**	0.46	0.12	0.21	0.34	0.24	0.22	0.04	0.04	0.1	0.01
**Serum 5**	0.61	0.22	0.29	0.37	0.25	0.26	0.06	0.06	0.14	0.03
**Serum 6**	0.47	0.18	0.25	0.32	0.27	0.29	0.03	0.06	0.28	0.03
**Serum 7**	0.33	0.11	0.12	0.24	0.1	0.09	0.07	0.04	0.08	0.05
**Serum 8**	0.5	0.24	0.04	0.13	0.07	0.05	0.03	0.03	0.02	0.04
**Serum 9**	0.38	0.26	0.28	0.27	0.04	0.02	0.19	0.21	0.1	0.06
**Serum 10**	0.8	0.42	0.49	0.55	0.42	0.42	0.13	0.23	0.46	0.13
**Serum 11**	1.35	0.66	0.87	1.27	0.69	0.43	0.44	0.63	0.17	0.07
**Serum 12**	0.73	0.45	0.46	0.44	0.28	0.25	0.15	0.17	0.12	0.1
**Serum 13**	0.54	0.29	0.23	0.26	0.14	0.12	0.06	0.07	0.06	0.02
**Serum 14**	0.54	0.42	0.19	0.23	0.01	0	0.15	0.15	0.01	0.01
**Serum 15**	0.72	0.3	0.46	0.45	0.29	0.26	0.06	0.16	0.12	0.01
**Serum 16**	0.46	0.35	0.34	0.34	0.13	0.13	0.21	0.22	0.07	0.09
**Serum 17**	0.84	0.41	0.7	0.69	0.42	0.38	0.33	0.38	0.23	0.04
**Serum 18**	0.36	0.19	0.3	0.24	0.15	0.2	0.12	0.1	0.14	0.05

**Serum 19**	0.34	0.2	0.26	0.25	0.21	0.29	0.27	0.18	0.11	0.05
**Serum 20**	0.35	0.23	0.27	0.25	0.2	0.17	0.15	0.15	0.09	0.05
**Total mean**	0.58	0.29	0.34	0.4	0.24	0.22	0.15	0.17	0.15	0.05
**Friedman's test (95% Confidence Interval) *p* value** < 0.0001										
**Dunn's *p* value**	-	<0.01	Not significant	Not significant	<0.0001	<0.0001	<0.0001	<0.0001	<0.0001	<0.0001
**SD**	0.26	0.13	0.2	0.26	0.16	0.14	0.11	0.14	0.12	0.033
**SEM**	0.06	0.03	0.04	0.06	0.04	0.03	0.03	0.03	0.03	0.01
**Median**	0.5	0.28	0.29	0.34	0.24	0.22	0.13	0.15	0.12	0.05
**Lower 95% CI**	0.4581	0.2304	0.2499	0.2770	0.1645	0.1595	0.09982	0.1036	0.09423	0.03005
**Upper 95% CI**	0.6989	0.3526	0.4341	0.5160	0.3175	0.2875	0.2022	0.2344	0.2058	0.06095

Supplementary Table S4. Demographic and clinical information of the olive pollen allergic patients used in the study.

Patient Number	Age/Sex	IgE olive pollen extract/olive pollen allergens (KU/L or OD_{492nm})	IgE to other pollens (KU/L)	IgE food allergens (KU/L)	IgE other allergens (KU/L)	Symptoms	Origin
1	11/F	nOle e 1 = 84.7	ND	Chickpea: 2.51<br>Almond: 1.12<br>Hazelnut: 5.10<br>Nut: 24.70<br>Pistachio: 7.60<br>Cashew: 2.07<br>Sole: 24.50<br>Hake: 54.70<br>Tuna: 24.30	*Alternaria alternata* = 0.78	RC and asthma	Córdoba
2	19/F	Extract = 26.7<br>nOle e 1 = 3.52<br>nOle e 9 = 6.14	ND	ND	ND	RC	Córdoba
3	30/M	Extract = 17.8	*Lolium perenne* = 20.3<br>*Parietaria judaica* = 17.2	ND	ND	Asthma	Madrid
4	8/F	Extract = 166	*Lolium perenne*: 1.58	ND	*Alternaria alternata* = 6.39<br>*Felis domesticus* = 2.46<br>*Canis lupus* = 160.00	RC and asthma	Córdoba
5	9/M	Extract = 56	*Lolium perenne* = 38<br>*Plantago lanceolata* = 26	ND	ND	Asthma	Madrid
6	22/M	Extract = 31.2	ND	ND	ND	RC and Asthma	Córdoba
7	30/M	Extract = 0.39 (OD492nm)	ND	ND	ND	RC and asthma	Córdoba
8	27/M	Extract = 23	*Lolium perenne* = 61	ND	ND	Asthma	Madrid

9	41/F	(*)Unknown but positive against the extract by blotting; negative to nOle e 1 and rOle e 7 by ELISA	ND	ND	rPru p 3 = 10.00<br>nBos d 5 = 2.52<br>nBos de 8 = 4.21<br>nBos d 4 = 5.22<br>Banana = 4.67<br>rJug r 3 = 4.03<br>Lettuce = 2.64<br>Kiwi = 3.15<br>Pineapple = 2.81	Asthma and food allergy	Córdoba
10	5/M	nOle e 1 = 127<br>nOle e 7 = 0.81<br>nOle e 9 = 92.20	rPhl p 1= 1.11	ND	ND	RC and asthma	Córdoba
11	12/F	Extract = 18.3	ND	ND	ND	RC and asthma	Córdoba
12	6/M	Extract = 55	*Lolium perenne* = 33<br>*Plantago lanceolata* = 24	ND	ND	Asthma	Madrid
13	7/F	Extract = 100	*Lolium perenne* = 100<br>*Fraxinus americana* = 18	ND	ND	Asthma	Madrid
14	22/M	Extract = 60	ND	ND	ND	ND	Córdoba
15	25/F	Extract = 27	ND	ND	ND	ND	Córdoba
16	6/M	nOle e 1 = 22.50<br>nOle e 7 = 11.00<br>nOle e 9 = 6.29	ND	Almond = 3.19<br>Nut = 7.75<br>Pistachio = 2.68	ND	ND	Córdoba
17	18/M	Extract = 0.11 (OD492nm)	ND	ND	ND	RC and asthma	Córdoba
18	20/F	Extract = 0.26 (OD492nm)	ND	ND	ND	RC and asthma	Córdoba
19	28/F	Extract = 53	ND	ND	ND	RC and asthma	Córdoba
20	13/F	Extract = 47	*Lolium perenne* = 8<br>*Plantago lanceolata* = 12	ND	ND	Asthma	Madrid

M, male. F, female. ND, not determined. RC, Rhinoconjunctivitis

Supplementary Table S5-1. Oligonucleotide sequences used to clone the DNA inserts encoding the Ole e 15-PPIA chimeras and the Ole e 15-derived peptides using HiFi and Gateway cloning technologies.

**Insert Fw Ch1**	ACCGTTTTCTTTGACATGACAATCGATGGTGAACCTGTTGGTCGGATCGTGATGGAA
**Vector Rv Ch1**	TGTCATGTCAAAGAAAACGGTAGGATTTGCCATATGGCTGTGATGATGATGATGATGGCTGCTG
**Insert1 Rv Ch4**	AAACTTTTCACCATAAATGCTTTTGCCACCGGTACCATTATGGCGAGTAAAATCGCCTCCCTGGCACATG
**Insert2 Fw Ch4**	AAAAGCATTTATGGTGAAAAGTTTGAGGATGAAAATTTCATCCTAAAGCACACCGGTCCTGGTATTCTCTC
**Insert Rv Ch5**	GCGCTCAATGGCTTCTACCACATAGAAACCCTCTTTTACTTGCCCAAACACCACGT
**Vector Fw Ch5**	GTGGTAGAAGCCATTGAGCGCGTTGGATCTCGCTCTGGAAAGACTGCAAAGCCAGTGGTGG
**Insert Rv Ch7**	GCAACCACCACTTTCTTTGCAGTCTTTCCAGAGCGAGATCCAACTTGCTCAATGGCTTTTA
**Vector Fw Ch7**	GCAAAGAAAGTGGTGGTTGCTGACTGTGGTCAACTCGAATAGGAATTCGATCC
**Insert Rv Ch8**	CCACAATCTGCGATAGTGATCTTTTTACTAGTTTTGCCGTTCCTAGATCCAACTTGCTCAATGGCTTTT
**Vector Fw Ch8**	ATCACTATCGCAGATTGTGGGCAGCTTGAAtagGAATTCGATCCGGCTGCTAACAAAGCCCGAAAGGAAGCTGAGT
**Insert Rv Ch9**	CCCAAACACCACGTGTTTCCCA
**Vector Fw Ch9**	GAAACACGTGGTGTTTGGGAAAGTGAAAGAAGGCATGAATATTGTGGAGGCCATGGAGCGCTT
**28aVector Fw**	GAATTCGATCCGGCTGCTAACAAAGCCCGAAAGGA
**Vector Rv Ole e 15**	AAAACCTTAGGATTTGCCATATGGCTGTGATGATGATGATGATGGCTGCTG
**Insert Rv Ole e 15**	TTAGCAGCCGGATCGAATTCCTAGGAGAGTTGACCACAGTCAG
**Insert Fw Ole e 15**	ATGGCAAATCCTAAGGTTTTCTT
**Peptide 1 Fw***	ATGGCCAATCCCAAAGTTTTCTTCGACATGACGATT
**Peptide 1 Rv***	TTACAGCTCCATGACAATCCGACCCACCGGCTGTCCACCAATCGTCATGTCGAAGAAAA
**Peptide 2 Fw**	TTTGCTGACGTTGTTCCACGGACGAGCGAGAACTTT
**Peptide 2 Rv**	TTACTTCCCCACCCCCTTCTCACCCGTGCAAAGTGCCCGAAAGTTCTCGCTCGTCCGTG
**Peptide 3 Fw**	TTTGCTGACGTTGTTCCACGGACGAGCGAGAACTTT
**Peptide 3 Rv**	TTACTTCCCCACCCCCTTCTCACCCGTGCAAAGTGCCCGAAAGTTCTCGCTCGTCCGTG
**Peptide 4 Fw**	CGGGCACTTTGCACGGGTGAGAAGGGGGTGGGGAAG
**Peptide 4 Rv**	TTAGAAGGCCGATCCCTTGTAATGTAAGGGTTTGCCTGACTTCCCCACCCCCTTCTCAC
**Peptide 5 Fw**	AAGTCAGGCAAACCCTTACATTACAAGGGATCGGCCTTC
**Peptide 5 Rv**	TTAGCCTCCTTGGCACATAAAATTAGGAATGACCCGATGGAAGGCCGATCCCTTGTAAT
**Peptide 6 Fw**	CATCGGGTCATTCCTAATTTTATGTGCCAAGGAGGC
**Peptide 6 Rv**	TTAGGACTCGCCCCCGGTGCCGTTCCCGGCTGTGAAATCGCCTCCTTGGCACATAAAAT

**Peptide 7 Fw**	GATTTCACAGCCGGGAACGGCACCGGGGGCGAGTCC
**Peptide 7 Rv**	TTACACGAAATTCTCGTCCGCAAATTTCGATCCGTAGATGGACTCGCCCCCGGTGCCGT
**Peptide 8 Fw**	ATCTACGGATCGAAATTTGCGGACGAGAATTTCGTG
**Peptide 8 Rv**	TTAAGCCATGGACAAGATCCCTGGGCCAGTATGTTTCTTCACGAAATTCTCGTCCGCAA
**Peptide 9 Fw**	AAGAAACATACTGGCCCAGGGATCTTGTCCATGGCT
**Peptide 9 Rv**	TTAAAAGAATTGGGACCCGTTTGTTCCTGGCCCTGCATTAGCCATGGACAAGATCCCTG
**Peptide 10 Fw**	AATGCAGGGCCAGGAACAAACGGGTCCCAATTCTTT
**Peptide 10 Rv**	TTATTTCCCGTCCAACCACTCCGTTTTGGCGGTACAGATAAAGAATTGGGACCCGTTTG
**Peptide 11 Fw**	ATCTGTACCGCCAAAACGGAGTGGTTGGACGGGAAA
**Peptide 11 Rv**	TTAATAGAACCCTTCGACTACCTGGCCAAATACGACGTGTTTCCCGTCCAACCACTCCG
**Peptide 12 Fw**	CACGTCGTATTTGGCCAGGTAGTCGAAGGGTTCTAT
**Peptide 12 Rv**	TTAGCTACCCGACCCGACCTGCTCGATGGCTTTTACCACATAGAACCCTTCGACTACCT
**Peptide 13 Fw**	GTGGTAAAAGCCATCGAGCAGGTCGGGTCGGGTAGCGGAAAGACCGCA
**Peptide 13 Rv**	TTAACTAAGCTGGCCGCAATCCGCCACAACTACAGGTTTTGCGGTCTTTCCGCTACCCGACCCGACCTGCT

* All oligonucleotides for peptide cloning included the 5'-sequences of the *Att*B-recombination sites (*ggggacaagtttgtacaaaaaagcaggcttc* for the forward (Fw) oligonucleotides, and *ggggaccactttgtacaagaaagctgggtc* for the reverse (Rv) oligonucleotides).

Supplementary Table S5-2. Oligonucleotide combinations used to produce the expression vectors containing the DNA inserts encoding Ole e 15-PPIA chimeras.

	**Insert 1**		**Insert 2**		**Linearized vector**	
	**INSERT 1 FW**	**INSERT 1 RV**	**INSERT 2 FW**	**INSERT 2 RV**	**VECTOR Fw**	**VECTOR RV**
**CHIMERA 1**	Insert Fw Ch1	Insert Rv Ole e 15	-	-	28aVector Fw	Vector Rv-Ch1
**CHIMERA 4**	Insert Fw Ole e 15	Insert1 Rv Ch4	Insert2 Fw Ch4	Insert Rv Ole e 15	28aVector Fw	Vector Rv Ole e 15
**CHIMERA 5**	Insert Fw Ole e 15	Insert Rv Ch5	-	-	Vector Fw Ch5	Vector Rv Ole e 15
**CHIMERA 7**	Insert Fw Ole e 15	Insert Rv Ch7	-	-	Vector Fw Ch7	Vector Rv Ole e 15
**CHIMERA 8**	Insert Fw Ole e 15	Insert Rv Ch8	-	-	Vector Fw Ch8	Vector Rv Ole e 15
**CHIMERA 9**	Insert Fw Ole e 15	Insert Rv Ch9	-	-	Vector Fw Ch9	Vector Rv Ole e 15
**CHIMERA 15**	Insert Fw-Ch1	Insert Rv Ch5	-	-	Vector Fw Ch5	Vector Rv Ch1
**CHIMERA 17**	Insert Fw-Ch1	Insert Rv Ch7	-	-	Vector Fw Ch7	Vector Rv Ch1

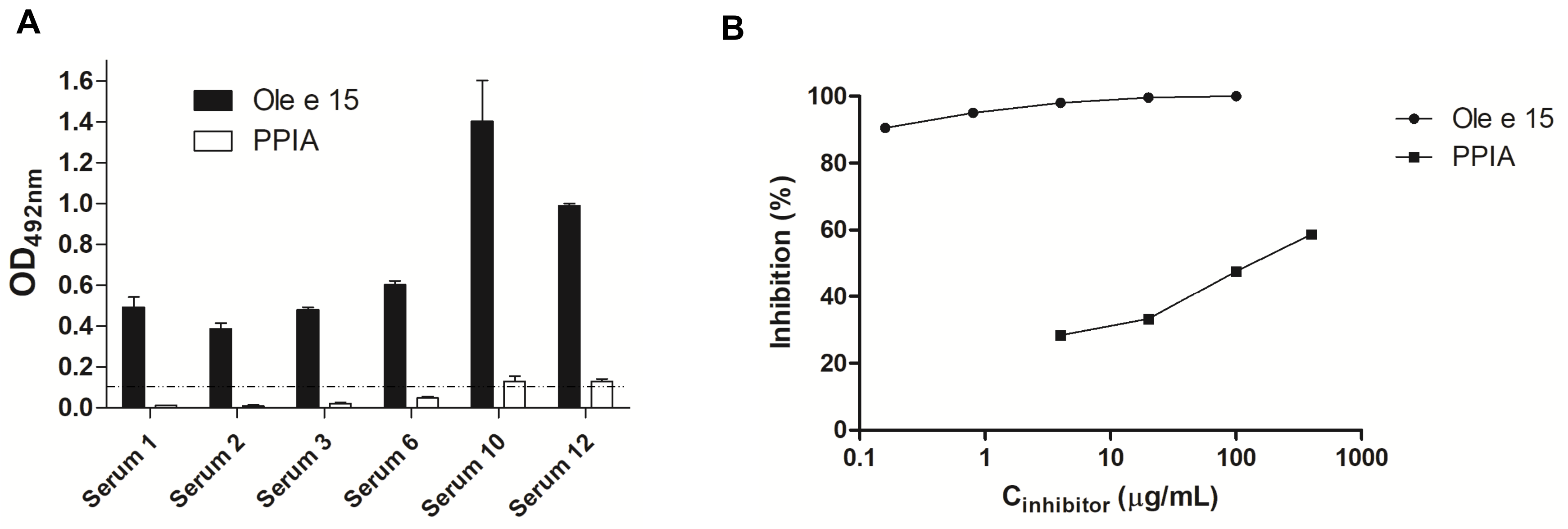

Supplementary Fig. S1.

Analysis of the *in vitro* IgE cross-reactivity between Ole e 15 and PPIA using six representative Ole e 15-allergic patients. (A) ELISA assessment of the IgE-binding to Ole e 15 and PPIA of the serum samples ten-fold diluted. Results are shown in $OD_{492\ nm}$ values (arbitrary units), *vertical lines* represent standard deviation (±SD, *error bars* for duplicates) and *dashed lines* represent the cut-off level of IgE-binding. (B) ELISA assessment of the inhibition of IgE-binding to immobilized Ole e 15 by means of serum preincubation with PPIA, and Ole e 15 as control inhibition.

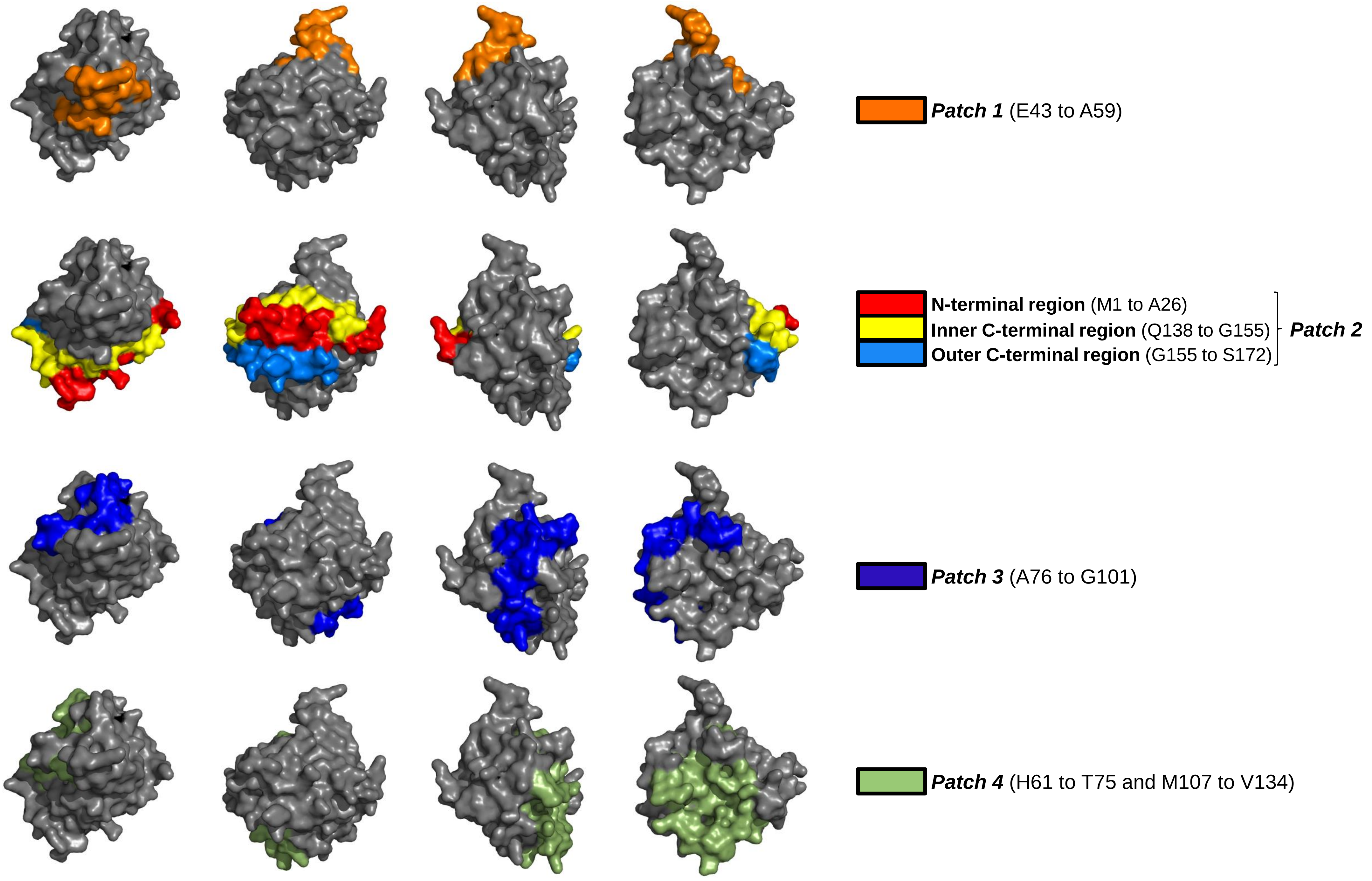

Supplementary Fig. S2

Different views of the modeled molecular surface of Ole e 15 showing the identified patches in different colors. *Patch 1* contains fourteen amino acids with a relative SASA value higher than 25% (E43, K44, G45, V46, G47, K48, S49, G50, K51, P52, H54, K56, G57, A59); *Patch 2* contains thirty two amino acids (M1, A2, N3, P4, K5, G13, G14, Q15, P16, V17, R18, Q138, V140, E141, F143, Y144, K147, A148, Q151, V152, S154, G155, S156, K158, A160, K161, P162, D167, Q170, L171, S172); *Patch 3* contains twenty four amino acids (A76, N78, G69, T70, G81, E83, S84, I85, Y86, G87, S88, K89, F90, A91, D92, E93, N94, F95, V96, K97, K98, H99, T100, G101); *Patch 4* contains twenty seven amino acids (H61, R62, N66, F67, C69, Q70, T75, N109, A110, G111, P112, T114, N115, S117, Q118, F119, T123, A124, K125, T126, E127, W128, L129, D130, G131, K132, H133). All 3D-models were visualized using PyMOL 2.3 (https://pymol.org/2/).

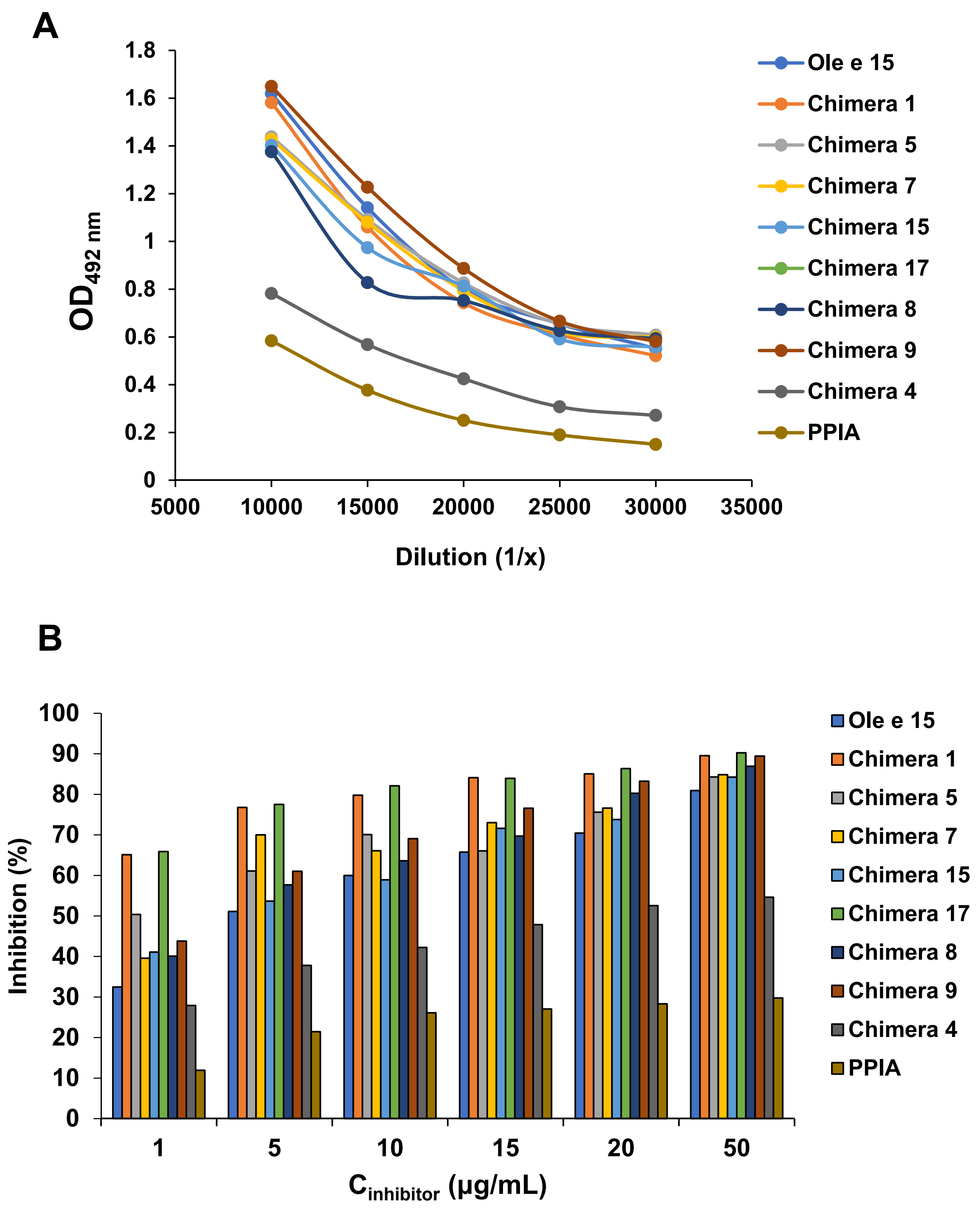

Supplementary Fig. S3

Polyclonal IgG-binding capacity of Ole e 15, PPIA and Ole e 15-PPIA chimeras. (A) Ole e 15-specific pAb titration curves obtained by ELISA against immobilized Ole e 15, PPIA and the chimeras. (B) Bar-graph showing the inhibition values of the Ole e 15-specific pAb-binding to immobilized Ole e 15 by means of preincubation with Ole e 15, PPIA and the chimeras.

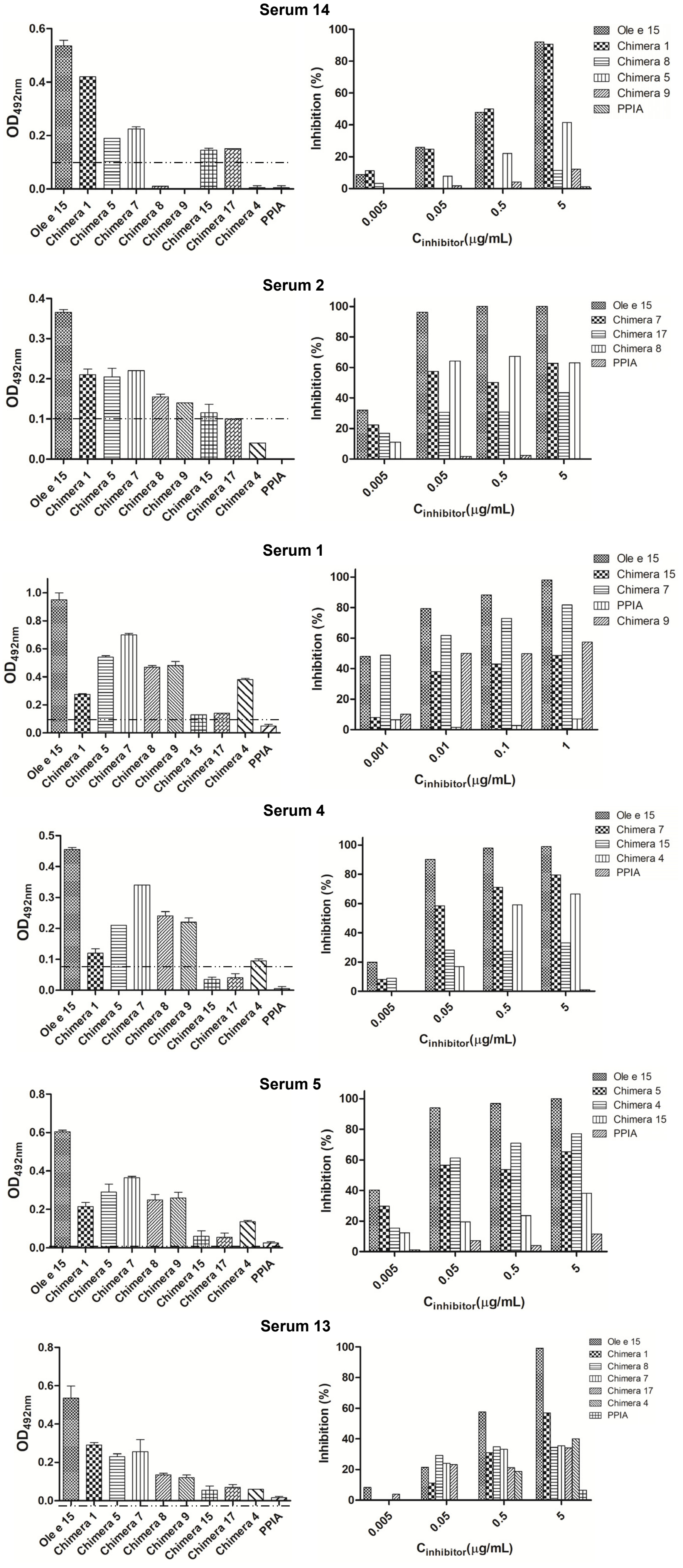

Supplementary Fig. S4.

Immunological characterization of the IgE-response to Ole e 15, PPIA and representative chimeras of six olive pollen allergic patients. Left panels show bar graphs representing the mean $OD_{492\ nm}$ values obtained by ELISA for the assessment of the IgE-reactivity (*vertical lines* represent standard deviation (±SD, *error bars* for duplicates), *dashed lines* represent the cut-off level of IgE-binding). Right panels show bar graphs representing inhibition values of IgE-binding to immobilized Ole e 15 assessed by ELISA after serum preincubation using indicated amounts of Ole e 15, PPIA and Ole e 15-PPIA chimeras as inhibitors.

A

```
                 Patch 2-Nt                                       Patch 1
Ole e 15     MANPKVFFDMTIGGQPVGRIVMELFADVVPRTSENFRALCTGEKGVGKSGKPLHYKGSAF 60
peptide1     MANPKVFFDMTIGGQPVGRIVMEL------------------------------------ 24
peptide2     ------------GGQPVGRIVMELFADVVPRTSENF------------------------ 24
peptide3     ------------------------FADVVPRTSENFRALCTGEKGVGK------------ 24
peptide4     ------------------------------------RALCTGEKGVGKSGKPLHYKGSAF 24
peptide5     -----------------------------------------------KSGKPLHYKGSAF 13
peptide6     ------------------------------------------------------------ 0
peptide7     ------------------------------------------------------------ 0
peptide8     ------------------------------------------------------------ 0
peptide9     ------------------------------------------------------------ 0
peptide10    ------------------------------------------------------------ 0
peptide11    ------------------------------------------------------------ 0
peptide12    ------------------------------------------------------------ 0
peptide13    ------------------------------------------------------------ 0

             Patch 4        Patch 3                              Patch 4
Ole e 15     HRVIPNFMCQGGDFTAGNGTGGESIYGSKFADENFVKKHTGPGILSMANAGPGTNGSQFF 120
peptide1     ------------------------------------------------------------ 24
peptide2     ------------------------------------------------------------ 24
peptide3     ------------------------------------------------------------ 24
peptide4     H----------------------------------------------------------- 25
peptide5     HRVIPNFMCQGG------------------------------------------------ 25
peptide6     HRVIPNFMCQGGDFTAGNGTGGES------------------------------------ 24
peptide7     ------------DFTAGNGTGGESIYGSKFADENFV------------------------ 24
peptide8     ------------------------IYGSKFADENFVKKHTGPGILSMA------------ 24
peptide9     ------------------------------------KKHTGPGILSMANAGPGTNGSQFF 24
peptide10    ------------------------------------------------NAGPGTNGSQFF 12
peptide11    ------------------------------------------------------------ 0
peptide12    ------------------------------------------------------------ 0
peptide13    ------------------------------------------------------------ 0

             Patch 4          Patch 2-Ct
Ole e 15     ICTAKTEWLDGKHVVFGQVVEGFYVVKAIEQVGSGSGKTAKPVVVADCGQLS 172
peptide1     ---------------------------------------------------- 24
peptide2     ---------------------------------------------------- 24
peptide3     ---------------------------------------------------- 24
peptide4     ---------------------------------------------------- 25
peptide5     ---------------------------------------------------- 25
peptide6     ---------------------------------------------------- 24
peptide7     ---------------------------------------------------- 24
peptide8     ---------------------------------------------------- 24
peptide9     ---------------------------------------------------- 24
peptide10    ICTAKTEWLDGK---------------------------------------- 24
peptide11    ICTAKTEWLDGKHVVFGQVVEGFY---------------------------- 24
peptide12    ------------HVVFGQVVEGFYVVKAIEQVGSGS---------------- 24
peptide13    ------------------------VVKAIEQVGSGSGKTAKPVVVADCGQLS 28
```

B

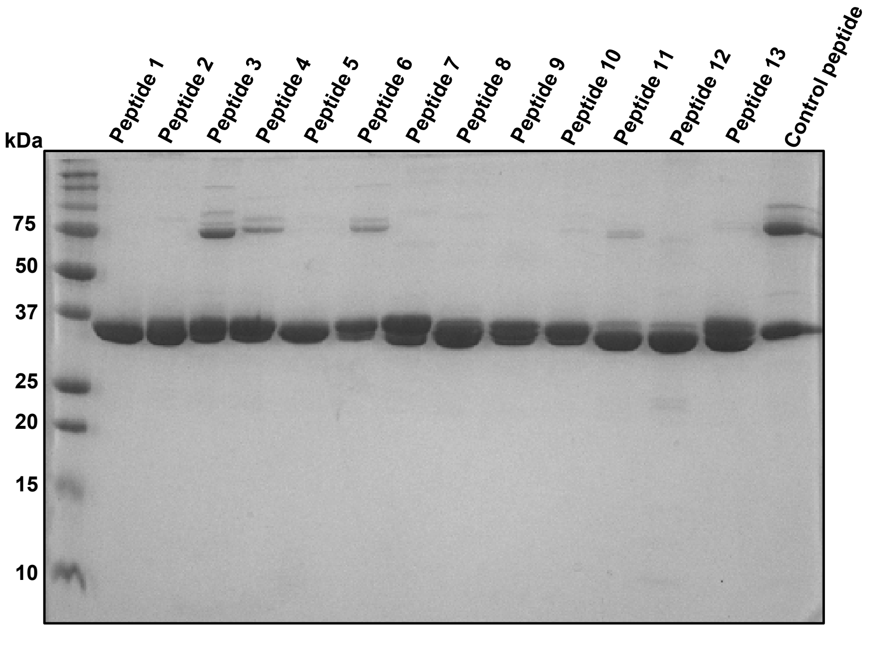

Supplementary Fig. S5

(A) Multiple sequence alignment between Ole e 15 and the designed peptides expressed as HaloTag-fused proteins. Sequences of the Ole e 15 surface patches are framed and shown in bold. (B) Coomassie Blue staining of 2 μg of the purified HaloTag-peptides and HaloTag with a random peptide after 15% SDS-PAGE under non-reducing conditions.

A

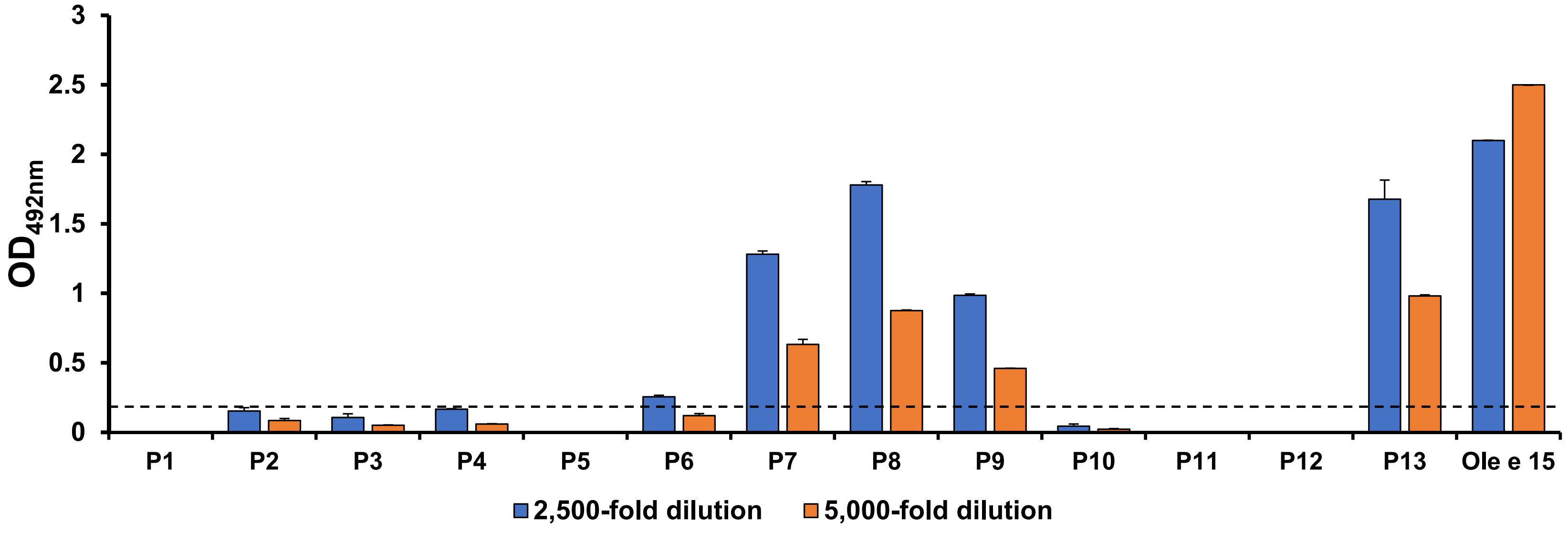

B

SERUM\INHIBITOR	Peptides 1 and 2 (*Patch 2,* N-terminal)	Peptides 4 and 5 (*Patch 1*)	Peptides 7 and 8 (*Patch 3*)	Peptides 12 and 13 (*Patch 2,* C-terminal)	Control	rOle e 15
**Serum 3**	3.16	63.11	0	0	0	100
**Serum 4**	0	0	0	0	0	100
**Serum 5**	2.89	4.91	0	5.05	0	100
**Serum 11**	0	0	0	0	0	100

Supplementary Fig. S6

(A) ELISA assessment of the IgG-binding capacity of HaloTag-fused Ole e 15-derived peptides. Ole e 15-specific pAb was 2,500 and 5,000-fold diluted. Results are shown as $OD_{492\ nm}$ values (arbitrary units). *Vertical lines* represent standard deviation (±SD, *error bars* for duplicates), the *dashed line* represent the cut-off level of IgG-binding. (B) ELISA analysis of the inhibition (%) of the IgE-binding to immobilized Ole e 15 after preincubation of serum samples with Ole e 15 or the indicated mix of HaloTag-fused peptides (2.5 µM each). 6×His-HaloTag protein with a random peptide was used as control.
